# Supplementary material for: Using Propensity Score Matching to Control for MRI Scan Quality
Source: bioRxiv. 2025 Sep 28:2025.09.26.678901. Preprint. [Version 1] doi: 10.1101/2025.09.26.678901 (PMC12485671; doi:10.1101/2025.09.26.678901)
Supplement: 1 [file NIHPP2025.09.26.678901V1-supplement-1.pdf]

bioRxiv preprint doi: https://doi.org/10.1101/2025.09.26.678901; this version posted September 28, 2025. The copyright holder for this preprint (which was not certified by peer review) is the author/funder, who has granted bioRxiv a license to display the preprint in perpetuity. It is made available under aCC-BY 4.0 International license.

# Supplementary Materials

**Table S1**

*MRI Acquisition Details for All Three Datasets*

| Dataset | Site | Image Acquisition | Make (model) | Voxel Size (mm) | Flip Angle (deg) | TR (ms) | TE (ms) |
|---|---|---|---|---|---|---|---|
| **ABIDE** | Caltech | 3D MPRAGE | Siemens Magnetom (TrioTim) | 1 x 1 x 1 | 10 | 1590 | 2.73 |
| | KKI | 3D FFE | Philips (Achieva) | 1 x 1 x 1 | 8 | 8 | 3.7 |
| | KUL | 3D FFE | Philips (Intera) | 0.98 x 0.98 x 1.2 | 8 | 9.6 | 4.6 |
| | MPG | 3D MPRAGE | Siemens Magnetom (Verio) | 1 x 1 x 1 | 9 | 1800 | 3.06 |
| | NYU | 3D MPRAGE | Siemens Magnetom (Allegra) | 1.3 x 1 x 1.3 | 7 | 2530 | 3.25 |
| | OHSU | 3D MPRAGE | Siemens Magnetom (TrioTim) | 1 x 1 x 1 | 10 | 2300 | 3.58 |
| | OLIN | 3D MPRAGE | Siemens Magnetom (Allegra) | 1 x 1 x 1 | 8 | 2500 | 2.74 |
| | Pitt | 3D MPRAGE | Siemens Magnetom (Allegra) | 1.1 x 1.1 x 1.1 | 7 | 2100 | 3.93 |
| | SBL | 3D FFE | Philips (Intera) | 1 x 1 x 1 | 8 | 9 | 3.5 |
| | SDSU | 3D SPGR | GE (MR750) | 1 x 1 x 1 | 45 | 11.08 | 4.3 |
| | SJH | 3D MPRAGE | Siemens Magnetom (Verio) | 1 x 1 x 1 | 8 | 1870 | 2.48 |
| | Stanford | 3D SPGR | GE (Signa) | 0.86 x 1.5 x 0.86 | 15 | 8.4 | 1.8 |
| | UCLA | 3D MPRAGE | Siemens Magnetom (TrioTim) | 1 x 1 x 1.2 | 9 | 2300 | 2.84 |
| | UM | 3D SPGR | GE (MR750) | 1.2 x 1 x 1.2 | 15 | 250 | 1.8 |
| | USM | 3D MPRAGE | Siemens Magnetom (TrioTim) | 1 x 1 x 1.2 | 9 | 2300 | 2.91 |
| | Yale | 3D MPRAGE | Siemens Magnetom (TrioTim) | 1 x 1 x 1 | 9 | 1230 | 1.73 |

bioRxiv preprint doi: https://doi.org/10.1101/2025.09.26.678901; this version posted September 28, 2025. The copyright holder for this preprint (which was not certified by peer review) is the author/funder, who has granted bioRxiv a license to display the preprint in perpetuity. It is made available under aCC-BY 4.0 International license.

| | | | | | | | |
|---|---|---|---|---|---|---|---|
| **COBRE** | MRN | Multi-Echo MPRAGE | Siemens Magnetom (TrioTim) | 1 x 1 x 1 | 7 | 2530 | 1.64, 3.5, 5.36, 7.22, 9.08 |
| **Healthy Aging** | Cornell | 3D SPGR | GE (MR750) | 1 x 1 x 1 | 7 | 2530 | 3.4 |
| | York | 3D FLASH | Siemens Magnetom (TrioTim) | 1 x 1 x 1 | 9 | 1900 | 2.52 |
